# Feasting on the ordinary or starving for the exceptional in a warming climate: Phenological synchrony between spongy moth (*Lymantria dispar*) and budburst of six European tree species

**DOI:** 10.1002/ece3.10928

**Published:** 2024-02-15

**Authors:** Yann Vitasse, Nora Pohl, Manuel G. Walde, Hannah Nadel, Martin M. Gossner, Frederik Baumgarten

**Affiliations:** ^1^ Swiss Federal Institute for Forest, Snow and Landscape Research WSL Birmensdorf Switzerland; ^2^ Southern Swedish Forest Research Centre Swedish University of Agricultural Sciences Lomma Sweden; ^3^ United States Department of Agriculture Animal and Plant Health Inspection Service, Forest Pest Methods Laboratory Buzzards Bay Massachusetts USA; ^4^ Department of Environmental Systems Science Institute of Terrestrial Ecosystems, ETH Zürich Zürich Switzerland

**Keywords:** budburst, chilling, phenological mismatch, phenology, spongy month

## Abstract

Global warming is affecting the phenological cycles of plants and animals, altering the complex synchronization that has co‐evolved over thousands of years between interacting species and trophic levels. Here, we examined how warmer winter conditions affect the timing of budburst in six common European trees and the hatching of a generalist leaf‐feeding insect, the spongy moth *Lymantria dispar*, whose fitness depends on the synchrony between egg hatch and leaf emergence of the host tree. We applied four different temperature treatments to *L. dispar* eggs and twig cuttings, that mimicked warmer winters and reduced chilling temperatures that are necessary for insect diapause and bud dormancy release, using heated open‐top chambers (ambient or +3.5°C), and heated greenhouses (maintained at >6°C or >10°C). In addition, we conducted preference and performance tests to determine which tree species the larvae prefer and benefit from the most. Budburst success and twig survival were highest for all tree species at ambient temperature conditions, whereas it declined under elevated winter temperature for *Tilia cordata* and *Acer pseudoplatanus*, likely due to a lack of chilling. While *L. dispar* egg hatch coincided with budburst in most tree species within 10 days under ambient conditions, it coincided with budburst only in *Quercus robur*, *Carpinus betulus*, and, to a lesser extent, *Ulmus glabra* under warmer conditions. With further warming, we, therefore, expect an increasing mismatch in trees with high chilling requirements, such as *Fagus sylvatica* and *A. pseudoplatanus*, but still good synchronization with trees having low chilling requirements, such as *Q. robur* and *C. betulus*. Surprisingly, first instar larvae preferred and gained weight faster when fed with leaves of *F. sylvatica*, while *Q. robur* ranked second. Our results suggest that spongy moth outbreaks are likely to persist in oak and hornbeam forests in western and central Europe.

## Graphical abstract

Our results show that under current winter conditions, the spongy moth egg hatch is well synchronized with the leaf emergence of a set of common European trees. However, under warmer winter conditions, we found an increasing mismatch in trees with high chilling requirement or photoperiodic sensitivity, such as European beech but still good synchronization with trees having low chilling requirement, such as pedunculate oak and hornbeam. Remarkably, *Q. robur* was the only species that advanced budburst to the same extent as *L. dispar* for all temperature treatments, demonstrating that both species do have similar temperature requirements for their ontogenetic development during winter and spring.

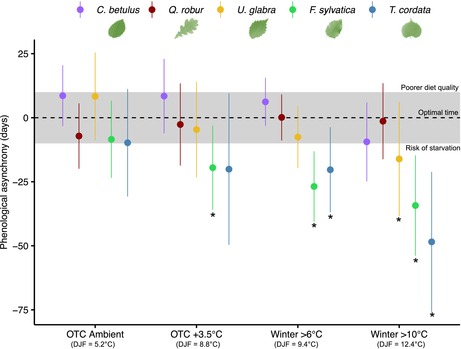

## 1 INTRODUCTION

Plant and animal species interact in ecosystems in many ways, and these biotic interactions maintain biodiversity and healthy ecosystem functioning (Ratzke et al., 2020; Slade et al., 2017). The fine‐tuned timing of reproduction and key events, such as spring leaf‐out for trees and egg hatching for insects, has been shaped by thousands of years of evolutionary history resulting in co‐existing and interacting species in a given ecosystem. Although species are able to adapt their phenology to new climatic conditions, the synchrony between interacting species is threatened by the rapidity of ongoing climate change (Visser & Gienapp, 2019). Global warming is affecting the seasonal life cycle of many organisms in the temperate zone, especially in spring, with, for example, earlier vegetation onset and insect development (Parmesan & Yohe, 2003; Vitasse et al., 2021). However, the magnitude of these phenological shifts varies substantially among interacting species and across trophic levels (Cohen et al., 2018; Parmesan, 2007; Thackeray et al., 2016; Vitasse et al., 2021), which may result in a temporal decoupling of trophic interactions, so‐called “phenological mismatches” (Beard et al., 2019; Duchenne et al., 2020; Kharouba et al., 2018; Reid et al., 2016; Thackeray et al., 2016). Phenological mismatches were suggested to be more common among antagonists (e.g., consumer‐resource) than among mutualists (e.g., plant‐pollinator) (Renner & Zohner, 2018; Visser & Gienapp, 2019). Tree–herbivore interactions are cases of antagonistic interactions that play an important role in the functioning of forest ecosystems (Weisser & Siemann, 2013). According to the Cushing match‐mismatch hypothesis (Cushing, 1969), the consumer, e.g., insect herbivore, attempts to synchronize the peak of its most energy‐demanding period with the peak of abundance and quality of its most consumed resource, e.g., tree leaves. Any change in this synchronization would result in a phenological “mismatch,” with positive consequences for the tree and negative consequences for the insect herbivore (Kharouba & Wolkovich, 2020). These high fitness costs for the insect will, however, likely also induce strong selection pressure, and the insect's high generation turnover rate may allow for rapid evolution if the population has sufficient phenological variability (McEvoy et al., 2012; Van Asch et al., 2007).

A large number of studies have shown that many temperate forest trees require a certain amount of time exposed to temperatures below a certain threshold (hereafter referred to as “chilling”) to release bud dormancy and that this amount of chilling determines the amount of heat (hereafter referred to as “forcing”) necessary to trigger budburst. Thus, it is expected that tree species with high chilling requirements will need progressively more forcing to leaf out due to the climate change‐induced decrease in chilling exposure during winter in many regions of the temperate climatic zone (Vitasse et al., 2018; Zhang et al., 2022). Tree species having either a high chilling requirement or high photoperiodic sensitivity such as European beech (Vitasse & Basler, 2013) show therefore lower phenological sensitivity to temperature increase than low chilling and/or photoperiodic insensitive tree species such as pedonculate oak (Baumgarten et al., 2021). This forcing‐chilling relationship and photoperiodic limitation have been proposed to explain the discrepancy between the actual and potential date of budburst under warmer temperatures in Europe (Fu et al., 2023) as well as the apparent decline in phenological sensitivity to temperature over the last decades (Fu et al., 2015), though this apparent decline may also result from non‐linearity of the temperature effect (Wolkovich et al., 2021).

Unlike trees, the requirement of chilling and forcing by folivorous (leaf‐feeding) insects to hatch and its consequence on insect fitness has been much less studied experimentally (but see Nielsen et al., 2022). Nevertheless, as for trees, shorter exposure to chilling conditions during winter diapause of many insects tends to increase the amount of forcing required for hatching (Tauber et al., 1986). The timing between the egg hatch of the herbivore and the host plant must be well synchronized to ensure the high fitness value of the insect (Hunter & Elkinton, 2000; Van Asch et al., 2007; van Asch & Visser, 2007). When herbivore larvae hatch well before leaf emergence, they are likely to starve while searching for food, resulting in high mortality and slow development. In contrast, when larvae hatch too late, the leaves have generally already started to decrease their nutritional quality by increasing the amount of defensive compounds such as phenolics and tannins, leading to reduced insect fitness, as demonstrated for spruce budworm (Lawrence et al., 1997), oak leafroller moth (Ivashov et al., 2002) and winter moth (Feeny, 1970). The strength of these effects could depend on the degree of specialization, with host specialists coping better with plant defences than generalists (but see Ali & Agrawal, 2012). The survival of the defoliator depends not only on its phenological synchrony with the host tree but also on climatic conditions (for example, frosts), natural enemies, tolerance to starvation, and the ability of the larvae to disperse to other plants. However, massive outbreaks of forest defoliating insects only occur when the herbivore is well synchronized with a high‐quality diet, i.e., when it hatches shortly before or during the budburst of its host tree (van Asch & Visser, 2007). This synchrony can be jeopardized by different sensitivity to climate warming between the defoliator and the host plant (Meineke et al., 2021). Yet only few experimental studies have quantified the phenological sensitivity of the host plant and associated herbivorous insects in response to winter and spring warming (see e.g. Kőrösi et al., 2018; Liu et al., 2011; Schwartzberg et al., 2014).

*The European spongy moth (Lymantria dispar dispar L.), whose fitness depends on the synchrony between egg hatch and leaf emergence of the host tree is a good example to study potential phenological mistmatches across trophic levels under warmer climate. Lymantria dispar* is a polyphagous defoliator species native to Europe and western Asia (Pogue & Schaefer, 2007). In North America, it is considered a pest species that has caused complete defoliation over large areas since its introduction in 1868 (Elkinton & Liebhold, 1990). Also, in Western and Central Europe, outbreaks are frequently recorded (Mirabel, 2021; Wulf & Graser, 1996) and are expected to increase with global warming (Logan et al., 2003). In Central Europe *L. dispar* is bound to warm and dry habitats such as sparse, sunny forests or forest edges (Nierhaus‐Wunderwald & Wermelinger, 2001). Population growth of *L. dispar* is favored by warm summers and prolonged drought. Outbreaks of *L. dispar* have been observed in different broad‐leaved forests, mainly in oak and chestnut forests, and more rarely in beech forests (Wulf & Graser, 1996). The most frequently infested tree species are oak, followed by hornbeam, beech, sweet chestnut, pome, and stone fruit (Boukouvala et al., 2022). On these species, spongy moth larvae develop fastest, show the lowest mortality and in the adult stage the females exhibit the highest fecundity and fertility. Conifers and herbaceous plants are used only during food shortage, except larch, which is a common host. The insect undergoes a prolonged obligatory pharate first instar diapause (Atay‐Kadiri & Benhsain, 2005; Tauber et al., 1990) and overwinters in egg masses deposited on tree trunks by the female imago. Low winter temperatures do not increase egg mortality significantly at temperate European latitudes, as eggs seem to tolerate temperature as low as −30°C (Fält‐Nardmann et al., 2018). Caterpillars usually hatch at the time of leaf flush and migrate to the crown after 2–3 days and start feeding. Caterpillar development lasts from 6 to 12 weeks after passing 5–6 (males) and 6–7 (females) larval instars. Although the caterpillars of *L. dispar* can feed on a multitude of species (Boukouvala et al., 2022), the first instar has the most specific host preference (Lechowicz, 1983) and typically feeds on elm, hornbeam, and especially oaks (Foss & Rieske, 2003; Raupp et al., 1988; Shields et al., 2003). Like many other defoliating insects that overwinter as eggs, *L. dispar* caterpillars can experience varying levels of diet quality, depending on the degree of timing between egg hatch and the appearance of leaves on trees within foraging distance, which strongly affects their survival and fitness (Foster et al., 2013; Stoyenoff et al., 1994). The best nutrition for the development of the first instar of *L. dispar* is found in freshly emerged leaves, which contain lower amounts of tannins and phenolics. Because nutritional quality generally decreases with leaf age (Hunter, 1993), the larval development rate and fecundity of the spongy moth decreases when hatching occurs too late after leaf‐out (Hunter & Elkinton, 2000; Martemyanov et al., 2015). At the same time, susceptibility to virus infection increases (Martemyanov et al., 2015) but decreases for certain bacterial pathogens (Martemyanov et al., 2016). However, it remains unclear whether the higher abundance of *L. dispar* in certain host trees is linked to a good synchronization of their hatching with the emergence of the host tree leaves irrespective of the climatic conditions, i.e., whether *L. dispar* eggs and the host tree have similar chilling requirement to break diapause/dormancy and similar forcing conditions to trigger hatch/budburst (Barbosa et al., 1979; Barbosa & Greenblatt, 1979; Raupp et al., 1988).

Here, we aimed to investigate how the timing between *L. dispar* hatching and leaf emergence of six common deciduous trees changes under four different winter conditions, using heated greenhouses and heated open top chambers. The experiment was complemented by a preference test of the first instar larvae for leaves of various tree species and a performance test by feeding the caterpillars with the leaves of each species separately to test whether the moth is better adapted to the leaves of tree species where it best synchronizes its hatching with budburst. *L. dispar* egg hatching is known to be highly sensitive to temperature and can be well modeled with degree‐hour accumulation (Waggoner, 1984), as well as the budburst timing of tree species with low chilling requirements such as pedunculate or sessile oak and hornbeam (Baumgarten et al., 2021). In contrast, some tree species, such as *F. sylvatica*, *T. cordata*, or *A. pseudoplatanus*, require a high chilling exposure to break winter dormancy and are therefore less directly responding to winter or spring warming (Baumgarten et al., 2021). Based on that, we formulated two hypotheses:

The hatching of the *L. dispar* eggs is well synchronized with the budburst of most of the study tree species under ambient conditions (sufficient chilling for all species). However, we expect an increasing mismatch under warmer winter with tree species having higher chilling requirements (*F. sylvatica*, *T. cordata* and *A. pseudoplatanus*).

First instar larvae show a stronger preference for tree species with which they are most phenologically synchronized and which, at the same time, provide them with the best diet for their development.

## 2 MATERIALS AND METHODS

### 2.1 Study species

We studied the hatching date of the polyphagous lepidopterous defoliator species, the spongy moth *Lymantria dispar dispar* L., in relation to the budburst of six native Central European broad‐leaved tree species under different winter conditions in controlled greenhouses and open top chambers at the research institute WSL, Birmensdorf, Switzerland. The six temperate broad‐leaved species are: *Acer pseudoplatanus* L., *Tilia cordata* Mill., *Fagus sylvatica* L., *Ulmus glabra* Huds., *Quercus robur* L., and *Carpinus betulus* L. These species often occur in forest communities in which outbreaks of *L. dispar* were observed (Boukouvala et al., 2022). In addition, they were selected due to their large variation in spring and autumn phenology. For example, the budburst timing of *F. sylvatica* is known to be sensitive to photoperiod and to be highly influenced by chilling temperature during winter and early spring (reviewed in Vitasse & Basler, 2013). In contrast, *Q. robur* is only marginally affected by photoperiod and has only low chilling requirements to break dormancy (Basler & Körner, 2012; Baumgarten et al., 2021; Laube et al., 2014). The other species may have intermediate requirements, with *U. glabra* and *C. betulus* being rather early flushing species in the study region and both having low forcing requirements, whereas *A. pseudoplatanus* and *T. cordata* show later spring flushing, likely due to higher chilling and forcing requirements (Baumgarten et al., 2021).

### 2.2 Experiment 1: Phenological synchronization between twig budburst and moth hatching

#### 2.2.1 Sampling and preparation of the twigs

Because young trees may not be representative of adult trees due to strong ontogenetic changes in phenological sensitivity (Vitasse, 2013), we used twig cuttings harvested from adult trees as they were found to respond very similarly to donor trees (Vitasse & Basler, 2014). For each tree species, we selected five healthy adult trees (>10 m height, >50 years) in a mature mixed forest near Zurich, Switzerland (47°21′22″ N, 8°27′35″ E; 550 m asl.). The mean annual air temperature of the site was 9.8°C and the mean annual precipitation was 1080 mm (1990–2020 mean recorded at the nearest climate station, Zurich Fluntern, 555 m asl., about 8 km away from the study site). The mean long‐term air temperature over the winter season (December to February) was 1.5°C, with a mean temperature of the coldest month (January) of about 1°C. On 27 November 2019, four twig cuttings at the tip of a branch were harvested for each of the five selected trees per species (total of 4 twigs × 5 individual trees × 6 species = 120 twigs) with a pole pruner. Twig cuttings were immediately put into moistened plastic bags in the field (to prevent desiccation) and within 1 h taken to the institute, where they were cut to a length of about 60 cm and placed in tap water at 5°C the same day. On 28 November, all twigs were pruned to a length of approximately 50 cm and placed into plastic boxes with deionized water to minimize bacterial development, as described in Baumgarten et al. (2021). Subsequently, they were subjected to four temperature treatments, with each of the five individuals (replicates) per species represented in each temperature treatment. To prevent vascular occlusion during the experiment, the base of each cutting was recut (by about 0.5 cm) and placed into fresh deionized water every week (greenhouse conditions; see below) or every second week (open top chambers).

#### 2.2.2 Preparation of the *L. dispar* eggs

For this experiment, the *L. dispar* eggs were sourced from the laboratory‐reared New Jersey Standard Strain (NJSS) colony maintained by the United States Department of Agriculture, Animal and Plant Health Inspection Service, Forest Pest Methods Laboratory (FPML) in Massachusetts, United States (Keena, 1993). This colony started with a mix of genotypes from Europe and follows a semi‐panmictic breeding protocol designed to avoid loss of genetic diversity. Nevertheless, the colony had been reared for 78 and 79 generations when the eggs were used in this study so that genetic diversity has presumably declined since the colony was established. Hatching eggs and larvae are held on a wheat‐germ‐based artificial medium (Bell et al., 1981) at 25°C, 70% RH, and 16:8 h (light:dark). Pupae are transferred to semi‐opaque containers where adults emerge, mate, and lay eggs on sheets of paper, which are harvested 42 days following pupa transfer. Diapausing *L. dispar* moth eggs were delivered from this laboratory on 6 November 2019 and kept outside the research institute, protected from rain and snow, until the start of the experiment. On 27 November 2019, as for the twig cuttings, five egg masses from five different *L. dispar* females, each consisting of around 600 eggs, were evenly split and assigned to the four temperature treatments (one replicate consists of ~50–250 eggs from one female, Table S1). Hence, all treatments held eggs from the same five females. Eggs were transferred to each temperature treatment and placed in open petri‐dishes that were placed within a larger one filled with a liquid detergent to prevent the larvae from escaping (Figure S1).

#### 2.2.3 Temperature treatments

**FIGURE 1:**
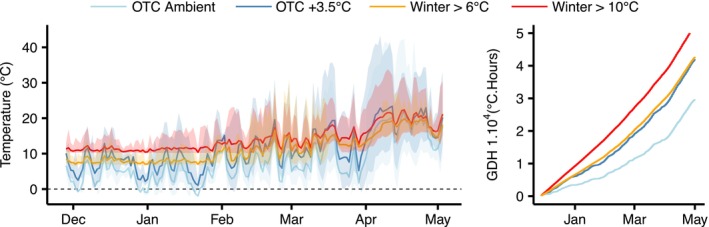
Air temperature and growing degree hours were recorded in the four different temperature conditions. Left: air temperature at twig bud height. The lines represent daily mean air temperature and shaded areas show daily minimum and maximum temperatures. Right: growing degree hours (GDH) above 0°C accumulated from the beginning of the experiment on 28 November 2019.

To simulate both realistic winter conditions in a future climate and reveal the effect of a lack of chilling on spring phenology, we simultaneously exposed twig cuttings and eggs to four temperature treatments. We used two “unrealistic” treatments using heated greenhouses where a temperature drop below either 6 or 10°C was prevented throughout the whole experiment to partially or almost totally exclude chilling temperatures that are assumed to break bud endodormancy and insect diapause. These two treatments are called hereafter “Winter >6°C” and “Winter >10°C” and correspond to low and very low chilling conditions over winter, respectively. The two other “more realistic” treatments consisted of two hexagonal glass‐walled open‐top chambers (3 m height, 6 m^2^ each), one experiencing ambient air temperature, whereas the other was maintained at 3.5°C above ambient air conditions using heating units (see Grossiord et al., 2022 for more details). We refer to them as “OTC Ambient” and “OTC +3.5°C”, respectively. We believe that a warming of 3.5°C in winter is a realistic prediction of what is likely to happen in the coming decades (Fischer et al., 2022). In fact, the mean annual temperature has already increased by about 2.6°C since 1960 at the study site (nearest climate station, Zurich Fluntern, 555 m a.s.l., about 8 km from the study site) and unusual warm spells in winter months exceeding past temperature norms by more than 3°C have already occurred at the study site. Throughout the experiment, temperatures were recorded at hourly intervals using temperature loggers (MX2203; Onset Computer Corp., Bourne, MA, USA). During the experiment, i.e., from 28 November 2019 to 2 May 2020 (latest budburst date recorded for sycamore maple twigs), the mean temperature in the different treatments was 7.8, 11.2, 11.4, and 14.0°C for the OTC Ambient, OTC +3.5°C, Winter >6°C and Winter >10°C, respectively, covering a gradient of 6.2°C (Figure 1). Similarly, during winter months (DJF), the temperature of the different treatments was 5.2, 8.8, 9.4, and 12.4°C, respectively. Temperature fluctuation in the open‐top chambers (OTC Ambient, OTC +3.5°C) was higher than in the greenhouse treatments (Winter >6°C, Winter >10°C) during winter (Figure 1) for which the absolute minimum temperature recorded throughout the experiment was 6.46°C (3 December 2019) and 10.01°C (13 February 2020), respectively (Figure 1). The treatment Winter >6°C and the OTC +3.5°C resulted in very similar accumulated growing degree hours (GDH) at the time of twig budburst or egg hatching of *L. dispar*, using 0°C as GDH threshold (Figure 1). However, assuming that chilling temperatures for trees are most efficient between 0 and 8°C (Hänninen, 2016), the treatment Winter >6°C resulted in lower chilling conditions than the treatment OTC +3.5°C after mid February (Figure S2).

#### 2.2.4 Phenology monitoring

Egg hatching and leaf emergence of twigs were monitored twice a week. At each monitoring, the first instar larvae were counted and removed and the phenological stage of the twigs was recorded using a five‐stage categorical scale (Vitasse et al., 2013): at stage 0 (dormant bud), no bud development was visible; at stage 1 (bud swelling), buds were swollen and/ or elongating; at stage 2 (budburst), bud scales were open and leaves were partially visible; at stage 3 (leaf‐out), leaves had fully emerged from the buds but were still folded, crinkled or pendant, depending on species; and at stage 4 (leaf unfolded), at least one leaf was fully unfolded. Because a lack of chilling may lead to erratic budburst, as found for either seedlings (Man et al., 2021) or cuttings (Baumgarten et al., 2021; Heide, 1993), we estimated the overall budburst performance of each twig by counting the percentage of buds that reached at least stage 2, i.e., budburst, 3 weeks after the first bud of a twig reached this stage, as well as how many twigs died during the experiment (i.e., twigs without any buds reaching stage 2 by the end of the experiment or that have not developed further than stage 2). The number of buds per twig varied across species due to species‐specific branch morphology with on average 5 buds for *A. pseudoplatanus*, 25 for *T. cordata*, 17 for *F. sylvatica*, 19 for *U. glabra*, 27 for *Q. robur* and 14 for *C. betulus*. Note that the percentage of buds per twig that reached budburst is very low for *Q. robur* irrespective of the treatments (on average 15%), which is also commonly observed in natural conditions due to a large amount of dormant reserve buds deployed in the event of leaf damage from herbivores or frost (Baumgarten et al., 2023).

### 2.3 Experiment 2: preference and performance tests for *L. dispar*

For the preference and performance tests, *L. dispar* eggs were obtained from the Bavarian State Institute of Forestry (LWF), Freising, Germany. This source was raised specifically to monitor their hatching phenology. Egg masses were first collected during the winter of 2020 in the forests of Germany's Lower Franconia region, between the city of Schweinfurt and Werneck, and then reared under laboratory conditions until new egg masses were laid. The egg masses were then exposed to ambient conditions and shipped to the WSL Research Institute on 27 October 2021, where they were again stored at ambient conditions until the start of the experiments.

The preference test took place on 23–24 March 2022 and consisted of placing 1.15 cm^2^ discs of freshly unfolded leaves (0–4 days after full leaf‐emergence) of each tree species in random order on wet absorbent paper in a petri dish (Figure S3), with five newly hatched larvae (0–2 days old) placed in the middle of the dish. This procedure was replicated twice for each of the five egg masses (i.e., 10 petri dishes in total). In another petri dish, leaf discs of all tree species, but without any larvae in them, were used as a control for the disc surface (moisture‐related shrinkage/expansion). All petri dishes were covered with a sheet of white paper to prevent excessive light from affecting larval behavior and kept in a climate chamber at 20°C and full illumination for 24 h (halogen lamps, photosynthetic photon flux density (PPFD) = 50 μmol m^2^ s^−1^ at bud height). The larvae were then removed, and discs were scanned individually using a flatbed scanner (EPSON Perfection V800 Photo; EPSON, Amsterdam) to determine the remaining leaf surface areas.

The performance test was conducted from 29 March 2022 to 4 April 2022 on unfed, newly hatched larvae (0–2 days old) from five egg masses of the same provenance. Larvae were exposed to freshly unfolded leaves (<4 days after the leaf unfolding stage) of a single tree species (6 tree species × 5 egg masses = 30 tubes). Larvae and leaves were put in a plastic tube with a permeable foam cap and kept at 20°C and constant light for 1 week using the same climate chambers as for the preference test. Desiccation of leaves and larvae was prevented by putting tubes upside down (i.e., cap side down) into a box with a 1–2 cm water layer. A paper sheet was placed on top of the tubes as protection from excessive light intensity (Figure S4). Depending on leaf size, two or three freshly unfolded leaves were added every other day to provide a constant source of fresh young leaves. For both experiments, fresh young leaves were obtained by regularly moving (February–March) potted saplings of each study species from ambient conditions outside into the climate chamber at 20°C. On 5 April (i.e., after 1 week), all five first instar larvae per tube were weighed individually using a microbalance accurate to 0.001 mg (Mettler Toledo Ltd. model AE 240, Greifensee, Switzerland), and the average fresh biomass per larva was used as an indicator for diet quality and performance (Barbosa & Greenblatt, 1979). Dead larvae were not weighed but counted as another indicator of leaf diet quality. In addition, as a control, five first instar larvae per egg mass were placed in the same type of tubes but without leaves and weighed after only 24 h, before death by starvation would occur. This control serves as reference for estimating the biomass gain of the caterpillars fed with leaves.

Experiment 1 and Experiment 2 used two different sources of *L. dispar*. To ensure that the American provenance used in Experiment 1 had a similar phenological sensitivity to temperature as the German provenance used in Experiment 2, we compared the hatching GDD requirements obtained in the four winter temperature treatments applied during the winter of 2020–2021 with the hatching GDD requirements of the German provenance exposed to different natural conditions during the winter of 2021–2022. Eggs of German provenance were placed in eight different forests in Switzerland (Table S2) and the hatching date was monitored by Swiss rangers (Project AGORA PhenoRangers, research grant no. CRAGP2_199833). Specifically, five eggs from each of the five egg masses of the same German provenance were placed in foam‐covered plastic tubes (similar to experiment 2) and attached to the north side of the trunk of a mature tree at a height of 2 m. A temperature logger (MX2203; Onset Computer Corp.) was also installed at a height of 2 m next to the tubes and recorded air temperature every hour. Although the winter 2020–2021 (experiment 1) was much warmer than the winter 2021–2022, degree hours at the time of hatching are in similar range between the German provenance installed in eight different sites and the US provenance under ambient controlled conditions (Table S2). In addition, a strong linear relationship was observed between winter temperature and degree‐hours required for egg hatching for both provenances combined, with no provenance‐related deviation (Figure S5). This result demonstrates that the two provenances show very similar phenological sensitivity to temperature so that the phenological synchrony between the trees and *L. dispar* (experiment 1) would show similar results if the more “local” provenance of *L. dispar* (German source) would have been used in experiment 1.

### 2.4 Data analysis and statistics

In experiment 1, the accumulated proportion of hatched larvae as a function of time (Day of the Year, DOY) was modeled in the different temperature treatments using generalized linear mixed effect models of the binomial family (logit link function) using the *glmer* function from the R‐package *lme4* (v. 1.1‐32, Bates et al., 2015) with egg mass origin as a random intercept. The hatching of *L. dispar* was also modeled for each egg‐mass separately using the *glm* function with a binomial family and time when 50% of the eggs of each egg mass hatched was extracted for comparison with twig budburst in further analyses.

For bud development in experiment 1, our subsequent analyses focus on stage 2 (budburst), as it can be easily differentiated from a dormant bud, whereas further development might be slowed down in twig cuttings compared to intact twigs (Baumgarten et al., 2021).

A linear mixed effect model with the function *lme* from the R‐package *nlme* (v. 3.1‐162, Pinheiro et al., 2023) was used to test the effect of the temperature treatment (fixed effect) on the budburst date for each tree species and the growing degree hours required for budburst/hatching including donor tree identity as a random intercept. To assess phenological (a)synchrony between egg hatch and twig budbreak, we refitted a linear mixed‐effects model with twig budburst date or caterpillar hatching date as the response variable, temperature treatment, and tree species as fixed effects with their interactions, and donor tree identity or egg mass as a random intercept. Budburst dates of *A. pseudoplatanus* were excluded from the analyses because a mortality higher than 50% was found in the two warmest temperature treatments. We accounted for the heterogeneity of variance between species and treatments in the variance structure of the model using the “weights” argument and the “varIdent” function. Phenological synchrony was assumed when values ranged between −10 and +10 days, with 0 day representing a perfect synchrony, negative values associated with a risk of starvation and positive values with poorer leaf nutritional quality (Hunter, 1993; Hunter & Elkinton, 2000). For experiment 2, i.e., the preference and performance tests, we also used linear mixed effect models with the function *lme* with egg masses as random intercept, to test (i) the differences in leaf disc surfaces between the ones in the presence of the larvae and the control discs (without larvae) after 24 h (preference test) and (ii) the differences in the fresh biomass gain per moth obtained after feeding them for 1 week with fresh leaves of each study tree species compared to the control without leaves (performance test). Normality and homoscedasticity of the residuals were visually checked in all applied models and were found to meet the assumptions of the models after using a log transformation of the independent variable when necessary. All analyses were performed using R Studio (v. 2022.07.2, R core team., 2020) and figures were created using *ggplot2* (v3.4.0). The package *car* (v. 3.1‐1 Fox & Weisberg, 2018) was used to run ANOVAs and the package *emmeans* (v. 1.8.7 Lenth et al., 2023) was used to extract marginal means and run post‐hoc tests using Tukey's honestly significant difference (HSD) in all models.

## 3 RESULTS

### 3.1 Hatching of *L. dispar* eggs in response to different temperature treatments

**FIGURE 2:**
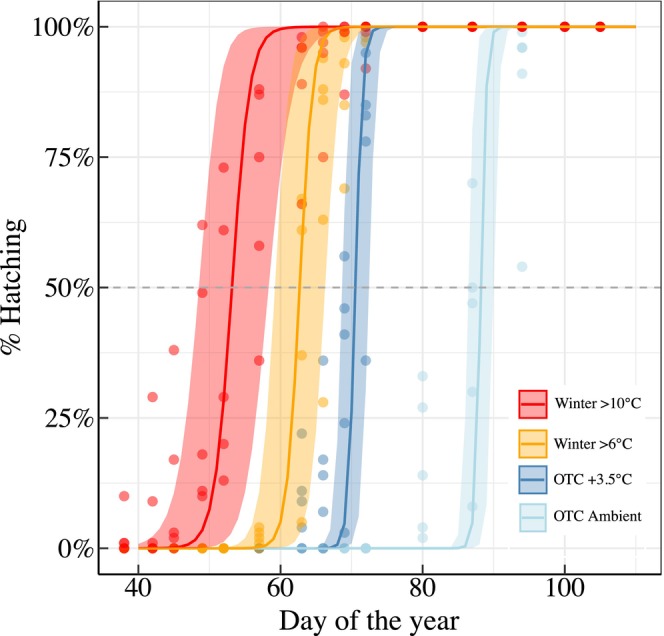
Progress of egg hatch over time in the different treatments. Egg‐hatching lines were predicted using generalized linear mixed‐effect models of the binomial family with egg mass (100–180 hatches per egg mass) as a random effect. Each dot corresponds to the accumulated percentage of hatched eggs per egg mass and treatments (observations). Shaded areas surrounding the lines correspond to 95% CI of the predicted values. The total number of eggs hatched was 739, 901, 693, and 502 for the treatments Winter >10°C, Winter >6°C, OTC +3.5°C, and OTC Ambient, respectively (see Table S1).

On average between 64 ± 4.5 and 182 ± 5.9 eggs (mean ± SE) successfully hatched per egg mass across treatments and between 502 ± 1.8 and 901 ± 3.0 (mean ± SE) per treatment across egg masses, with a total of 2835 hatched larvae (Figure S6 and Table S1). Overall, temperature treatments significantly affected the timing of egg hatching (*p* < .001, Table S3). The warmest winter condition (winter >10°C) for instance advanced egg hatching more than 1 month compared to the coldest winter conditions (OTC Ambient, Figure 2). Thus, 50% hatching was reached at the day of the year (DOY) 53.6 ± 1.8 (mean DOY ± SE), 63.1 ± 0.7, 69.1 ± 0.8, and 87.4 ± 1.3 for the treatments winter >10°C, winter >6°C, OTC +3.5°C and OTC Ambient, respectively (Figure 2). No significant differences were found between egg masses (*p* = .052, Table S3) which responded similarly to the temperature treatment (*p* = .893, Table S3 and Figure S7).

### 3.2 Budburst timing of the deciduous trees

**FIGURE 3:**
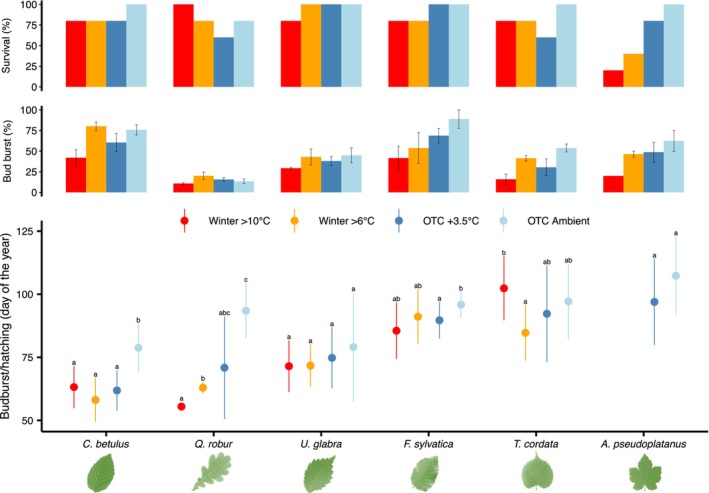
Budburst timing of twigs in the different treatments along with the survival rate and budburst success. Dots represent marginal means predicted from the linear mixed effect model for the date of budburst ±95% CI for twigs of the study species using donor tree as a random effect. Within species, means denoted by a common letter are not significantly different according to the Tukey test at the 5% level of significance. Note that the marginal means of *Acer* under Winter >10°C and Winter >6°C are not displayed due to insufficient survival (<50%). Vertical bars represent the survival rate of the twigs (% of twigs reaching stage 2) and budburst success per twig (mean ± SE).

Overall, temperature treatment significantly affected the timing of budburst and species responded differently to the temperature treatment as revealed by the significant interaction between species and temperature treatment (*p* < .001, Table S4) At ambient temperature (OTC Ambient), *C. betulus* and *U. glabra* were the first species to budburst (Mean DOY ± SE, 78.8 ± 3.9 and 79.1 ± 6.2, respectively), followed by *Q. robur* (93.5 ± 3.8), *F. sylvatica* (95.8 ± 1.8), and *T. cordata* (97.2 ± 4.3), and finally *A. pseudoplatanus* (107.0 ± 4.9, Figure 3). Overall, all species tended to advance budburst under warmer winter conditions, but the sequence of budburst between species was altered (Figure 3; Figure S8). For instance, while *C. betulus* and *U. glabra* ranked first and second in ambient condition, they ranked second and third in the Winter >10°C treatment, while *Q. robur* ranked first in the Winter >10°C treatment but only third in ambient conditions (Figure S8). *Q. robur* was the species exhibiting the strongest advancement of its budburst timing under warmer conditions, i.e., 39 days earlier at Winter >10°C compared to the ambient conditions, becoming the first tree species to budburst in this treatment (Figure 3). In contrast, a difference of only 9–10 days was observed between these two most contrasting treatments for *U. glabra* and *F. sylvatica*. Remarkably, *T. cordata* showed later budburst at Winter >10°C (102.0 ± 6.7) compared to all other treatments including ambient conditions (Figure 3). Overall, twig survival and budburst success declined with warmer winter conditions, which was particularly pronounced in *A. pseudoplatanus* and *T. cordata* and to a lower extent in *F. sylvatica*. In contrast, survival remained high (>60%) in *Q. robur*, *U. glabra*, and *C. betulus* under warmer winter conditions. Budburst success was lowest at Winter>10°C for all species and tended to increase under cooler winter conditions (Figure 3).

### 3.3 Budburst timing of the trees in relation to *L. dispar* egg hatch

**FIGURE 4:**
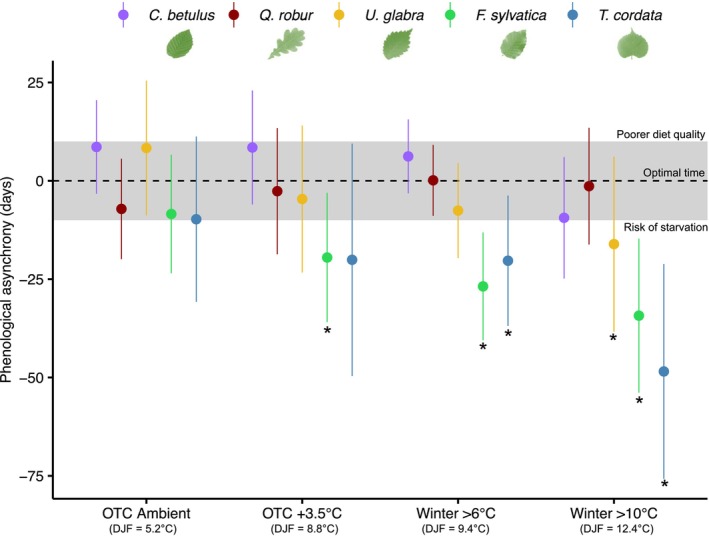
Phenological asynchrony between the hatching date of *Lymantria dispar* and budburst of the study tree species. Dots represent marginal means differences predicted from the linear mixed effect model between the 50% hatch date of *L. dispar* eggs and the date of twig budburst of the study species ±95% CI using donor tree or egg masses as a random effect. The dotted line represents a perfect match, and the gray area was defined as the optimal period. Positive values outside of the gray area correspond to poorer diet quality due to too late hatching (phenological asynchrony < +10 days), while negative values outside of the gray area correspond to risk of starvation due to too early hatching (phenological asynchrony < −10 days). These threshold values were defined according to Hunter (1993), and Hunter and Elkinton (2000). Stars indicate a significant difference between the egg hatching and budburst.

Overall the mixed effect model revealed a significant effect of the species, temperature treatment, and a species‐treatment interaction on budburst/hatch timing, indicating that species did not respond similarly to increasing temperature (Table S5). At ambient conditions, the timing of *L. dispar* egg hatch coincided fairly well with the budburst timing of all investigated tree species; it was slightly later than *C. betulus* and *U. glabra* and slightly earlier than all other tree species (Figure 4). However, under warmer winter conditions the timing of *L. dispar* egg hatching fell within the leaf emergence window of only *C. betulus*, *Q. robur* and to a lesser extent *U. glabra*, while egg hatching in treatments where the temperature was kept warmer than 6 or 10°C was significantly earlier than budburst of *F. sylvatica* and *T. cordata*, introducing a high risk of starvation (Figure 4). Egg hatching was also significantly earlier than the budburst of *F. sylvatica* under 3.5°C warmer conditions.

### 3.4 Feeding preference and performance tests of *L. dispar*

**FIGURE 5:**
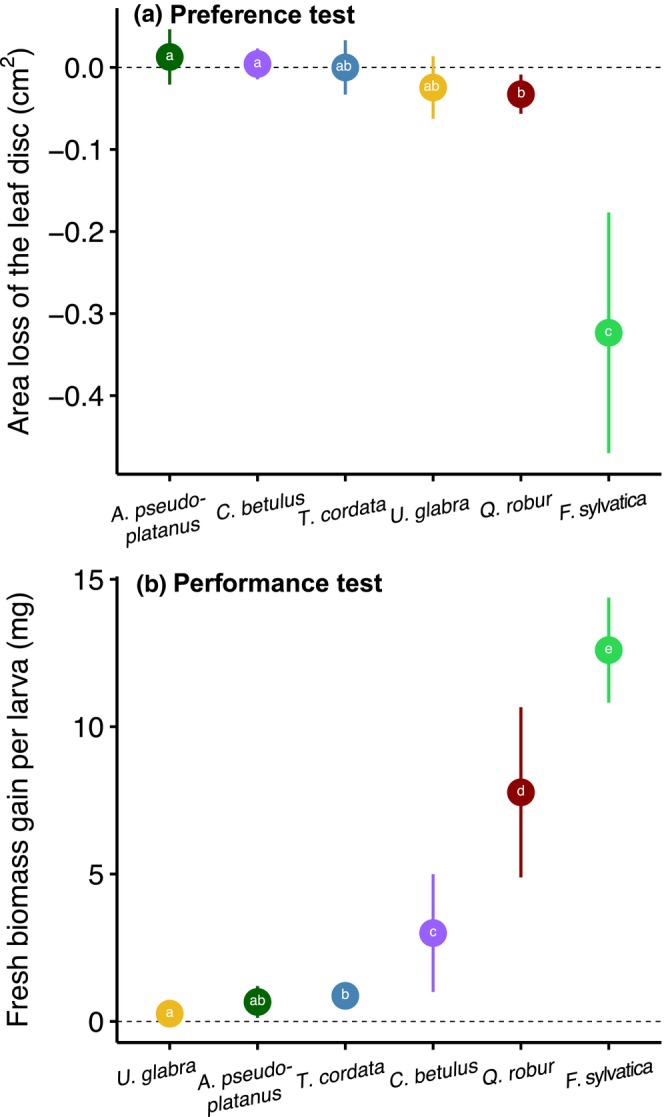
Preference and performance by first instar larvae on host tree species. (a) Leaf surface area consumed in tests to assess first instar larval feeding preference on different tree species and (b) biomass gained in performance tests of first instar larvae on each host species compared to the control. Preference tests were conducted for 24 h and performance tests for 1 week. Dots represent marginal means ±95% CI predicted from the linear mixed effect model using the source of egg mass as random effect. Means marked with a common letter are not significantly different by the Tukey test at the *5*% level of significance performed for each species.

The preference test performed on first instar larvae of *L. dispar* for 24 h at 20°C showed significant differences between tree species (Anova, *χ* ^2^ = 65.7, *p* < .001), with a clear preference by larvae for *F. sylvatica* leaf discs, which lost on average 0.323 cm^2^ (−26.0%) of leaf area compared to the control leaf discs, followed by *Q. robur*, which lost only 0.033 cm^2^ (−2.9%). No significant difference was found in any of the other species compared to the control leaf discs (Figure 5a).

The performance test on newly hatched larvae showed a significant difference in biomass gain between the different species (Anova, *χ* ^2^ = 2135, *p* < .001), with a higher biomass increase when larvae were fed with young leaves of *F. sylvatica* (+12.6 mg, i.e., +2603%), followed by *Q. robur* (+7.8 mg ±1.0, i.e., +1611%) and *C. betulus* (+3.0 mg, i.e., +620%), compared to the control larvae that were not fed with any leaves for 24 h. In contrast, larval biomass increased only slightly when exposed to *A. pseudoplatanus*, *U. glabra* or *T. cordata* leaves and differed only marginally from the control (Figure 5b). Overall mortality was 18.2%, but varied greatly according to diet, with a higher mortality rate for these latter species (>24%) and the lowest rate for *Q. robur* and *F. sylvatica* (<4%, Table S6). Thus, first instar larvae of *L. dispar* would starve rather than feed on *U. glabra*, *A. pseudoplatanus*, or *T. cordata*.

## 4 DISCUSSION

Different phenological shifts between interacting organisms are expected to occur under rapid global warming, due to differences in temperature sensitivity, which can result in the so‐called phenological mismatches. Yet only a few studies have attempted to investigate how synchronization between interacting species may alter in response to global warming experimentally. Our results show that under current conditions the hatching of *L. dispar* is well synchronized with the budburst of all studied tree species. However, in line with our hypothesis H1, warmer winter temperatures resulted in an increasing mismatch between the hatch of *L. dispar* and the budburst date of temperate trees with higher chilling requirement to break winter bud dormancy (*F. sylvatica*, *T. cordata*, and *A. pseudoplatanus*), whereas the synchronization persists with tree species having lower chilling requirement, and consequently higher phenological sensitivity to temperature increase (*Q. robur*, *C. betulus*, *U. glabra*). Remarkably, *Q. robur* was the only species that advanced budburst to the approximately same extent as *L. dispar* for all temperature treatments, demonstrating that both species do have similar temperature requirements for their ontogenetic development during winter and spring. Surprisingly, first instar larvae of *L. dispar* did not prefer host tree species with high‐temperature sensitivity as expected (H2 hypothesis). Instead, we found that larvae clearly preferred and performed better (survival and biomass) on the young leaves of *F. sylvatica*, which has a high chilling requirement and photoperiodic sensitivity and was not synchronized with the phenology of the moth under milder winter conditions, even at +3.5°C warmer conditions. Nevertheless, larval survival rates and mass gain were still high when feeding on leaves of the better synchronized *Q. robur*.

### 4.1 The phenological ranking among tree species is linked to their chilling and heat requirement

Warmer winter conditions significantly altered the sequence of budburst between the different tree species studied, due to their different sensitivities to forcing, chilling, and photoperiod. Similar chilling‐related rank changes in the sequence of leaf‐out timing in response to warming have been found in other studies (Flynn et al., 2018; Laube et al., 2014; Roberts et al., 2015). Here, we found that 3.5°C warming in a typical mid‐European climate (Zurich) advanced leaf‐out timing in all species. However, further warming resulted in different response groups that either (i) continued phenological advancement (*Q. robur*), (ii) stopped at the earliest budburst date limit (*U. glabra*, *C. betulus*, and *F. sylvatica*), or (iii) delayed budburst timing (*T. cordata*, and presumably *A. pseudoplatanus*). In other words, the additional thermal energy received by the latter group of species could not offset the lack of chilling, resulting in a reverse trend of budburst timing and a decrease in bud vitality. This is also reflected in the amount of heat accumulated at the time of budburst which increased more with warming for species requiring longer chilling exposure for dormancy release (*T. cordata* or *F. sylvatica*) than for species with lower chilling requirements (*Q. robur* or *C. betulus*, Figure S9). More importantly, budburst success and survival support this grouping, with the greatest declines in the warmest treatment occurring in species with higher chilling requirements, as was found by Baumgarten et al. (2021), with *Q. robur* not affected and *A. pseudoplatanus* barely surviving because very few buds could open and develop. The limited advance of budburst in the warmest treatments found for *U. glabra*, *C. betulus* and *F. sylvatica* may also indicate a critical influence of chilling and/or photoperiod. It is likely that bud development of *F. sylvatica* in the warmest treatment was also slowed by the shorter photoperiod in early spring, as this species is known to be sensitive to photoperiod (Vitasse & Basler, 2013) and its spring phenology is generally less responsive to warming than that of other species (Fu et al., 2019; Grossiord et al., 2022; Murray et al., 1989; Walde et al., 2022). In contrast, previous experimental studies showed no evidence of the influence of photoperiod on the budburst timing in *U. glabra* and *C. betulus* (Basler & Körner, 2012; Ghelardini et al., 2010; Laube et al., 2014), suggesting that the limited advance of spring phenology in the warmest treatment was entirely due to insufficient chilling in these two species. It is important to note that the percentage of bud burst appeared to be a much more sensitive and relevant indicator for studying the leaf‐out performance under limiting chilling conditions than leaf‐out timing of the first bud.

### 4.2 *Lymantria dispar* is more synchronized with *Q. robur* and *C. betulus*

Our results demonstrate that the hatching of *L. dispar* larvae closely follows the budburst timing of *Q. robur* and *C. betulus*, irrespective of climatic conditions. We suggest that these tree species and *L. dispar* have very little chilling requirements to break winter dormancy/diapause and are not sensitive to photoperiod, and therefore can respond quickly to warmer temperature. Our results corroborate recent herbarium findings showing that plant species with high‐phenological temperature sensitivity are more damaged by defoliating insects in warmer years than plants with low‐temperature sensitivity (Meineke et al., 2021). However, due to asymmetric temperature responses, other tree species that currently leaf out before the hatching of *L. dispar* may soon enter the optimal time window of the moth as climate continues to warm. Similarly, as *L. dispar* migrates farther to northern latitudes (Ponomarev et al., 2023), its phenology could match that of tree species that were too late in southern latitudes, as it has been recently found for the eastern spruce budworm with black spruce in North America (Bellemin‐Noël et al., 2021).

Spring hatching of caterpillars overwintering as eggs should be well synchronized with available resources to avoid starvation (resulting from premature hatching) or suffering from poorer leaf quality as polyphenols and tannins increase with leaf age (Erelli & Elkinton, 2000; Hunter & Elkinton, 2000). In fact, first instar of *L. dispar* larvae is susceptible to starvation if they do not find suitable host leaves within 5–10 days, depending on temperature, with higher tolerance under colder conditions (Hunter, 1993). The high mortality rate of larvae that hatch too early and the low weight gain and resulting lower fecundity of larvae that hatch too late result in high selection pressure toward a hatching date that is well synchronized with the phenology of the host tree (Van Asch et al., 2007). Hence, defoliation hotspots generally coincide with optimal phenological matching between insect and host tree with small deviations in leaf‐out timing modifying the degree of damage (Bellemin‐Noël et al., 2021; Jones & Despland, 2006; Raupp et al., 1988). This suggests that *Q. robur* and *C. betulus* will remain the tree species most susceptible to *L. dispar* outbreaks in a warming climate, while outbreaks in other important host species such as beech will become less likely. Note that the high synchrony between *Q. robur* and *L. dispar* could also be partially driven by the rough, dark tree trunks that offer good camouflage for *L. dispar* egg masses or first instar larvae against natural predators compared to other host trees such as beech or poplar with a smoother, lighter trunk (Roden et al., 1992).

### 4.3 Performance and preference of first instar larvae for different tree species

Our results show a significant preference of first instar larvae for *Q. robur*, *F. sylvatica*, and *C. betulus*, while they hardly feed on *A. pseudoplatanus*, *U. glabra*, and *T. cordata*. Similar preferences have been observed for related tree species in the U.S.A. (Barbosa et al., 1979; Lechowicz & Jobin, 1983), illustrating the close phylogenetic relationship between trees and the chemical constituents of their leaves. Although *L. dispar* can feed on a multitude of tree species at different stages of larval development, first instar larvae are much more selective, making the timing of egg hatch with the emergence of leaves of the preferred tree species a decisive factor in the development of an outbreak (Despland, 2018). In addition to the preference of *L. dispar* larvae for *F. sylvatica* leaves, larvae in our experiment achieved the highest weight with this food, suggesting freshly emerged *F. sylvatica* leaves have a high food quality. However, *L. dispar* larvae are poorly synchronized with *F. sylvatica* under warmer winter conditions, likely due to the high dependence of *F. sylvatica* on chilling and photoperiod. Outbreaks of *L. dispar* have been rarely observed in European beech forests over the past decades (McManus & Csóka, 2007), though under sufficient chilling *L. dispar* eggs should be quite well synchronized in some areas of Europe. Our results suggest that outbreaks could be possible in beech‐dominated forests under sufficient chilling in winter regarding their phenology but the absence of outbreaks in such forests might be due to other reasons such as natural enemies or not protected areas for the nest.

*Quercus robur* phenology was well synchronized with the hatching of *L. dispar* larvae in all tested climatic conditions, and the diet quality of young *Q. robur* leaves was also high. Therefore, our results suggest that the presence of *Q. robur* in the forest stand may favor the survival of *L. dispar* instars. In later larval development, *L. dispar* becomes less specific and can feed on many species, even conifers, so the presence of *Q. robur* may facilitate outbreaks of the moth in various forests. Preference for species of the *Quercus* and *Carpinus* genera during the forest regeneration phase in *F. sylvatica* stands may therefore increase the likelihood of future *L. dispar* outbreaks given similar pressure for natural enemies.

The suitability of various other tree species for *L. dispar* in terms of phenology and adaptation to a warmer climate should be further explored, especially with respect to tree species that are potentially better adapted to the future climate. For example, *L. dispar* outbreaks on Spanish chestnut (*Castanea sativa* Mill.) in southern Switzerland have been frequently reported and would merit further investigation.

### 4.4 Limitations of the study and knowledge gaps

Our experimental study allows us to disentangle tree‐related cues that are correlated under natural conditions, such as forcing and chilling effects. This in turn allows us to better predict future phenological changes than studies relying solely on long‐term phenological observations (Wolkovich et al., 2023). However, like many experimental studies on phenology, our study has some limitations.

First, the small number of replicates used for twig cutting certainly does not represent the large variability in budburst dates typically found in tree stands (Delpierre et al., 2020), and weakens the statistical robustness of the results, especially under low‐chilling conditions where some twigs show no budburst at all. However, the results found here are very consistent with previous experimental studies on tree phenological changes using the same species (Baumgarten et al., 2021; Laube et al., 2014) and we are therefore confident that the results are reliable.

Second, possible genetic adaptations to different climatic conditions could have influenced the timing of egg hatching with the emergence of tree leaves, since twigs were sourced from local trees, while moth eggs were supplied from a long‐established laboratory colony for experiment 1. However, there is no evidence of metabolic adaptation to colder environments between northern and southern populations of *L. dispar* in the U.S. (May et al., 2018) and the eggs used in our study were produced in a process that favors the retention of genetic diversity (e.g., no selection due to food shortage). In addition, we compared the amount of degree hours required for hatching between the US provenance of *L. dispar* used in experiment 1 and the German provenance used in experiment 2 after placing the latter provenance at eight different locations in Switzerland. The German provenance showed similar growing degree hours to hatch as the US provenance under similar winter temperature conditions, but values increase linearly with winter warming without any deviation related to the provenances (Figure S5).

Third, our different warming treatments yielded large differences in temperature fluctuations with much less fluctuations when temperature was maintained above 6 or 10°C in the greenhouses during winter than in ambient and +3.5°C open top chambers, which may have affected insect or plant development (Holyoak & Wetzel, 2020). However, evidence for a difference between fluctuating and constant temperature at the time of moth egg hatching or tree budburst is lacking. For example, budburst of fully chilled birch (*Betula pubescens*) was studied under six temperature regimes either fluctuating around the mean or using constant temperatures in climate chambers, and no difference was found in the thermal accumulation at the time of budburst between plants receiving constant or fluctuating temperatures (Myking, 1997). Similarly, no significant differences in the hatching of the diamondback moth (*Plutella xylostella*) were found between constant temperature treatment and treatments with moderate temperature fluctuations (Xing et al., 2015). However, an effect begins to appear under large temperature fluctuation likely due to the nonlinearity of insect development to temperature, i.e., that large temperature fluctuation includes temperatures that accelerate or slow down development (Holyoak & Wetzel, 2020; Worner, 1992). Consistent with these previous findings we found a similar amount of growing degree hours accumulated at the time of hatching or budburst in the two treatments OTC 3.5°C and Winter >6°C (Figure S9) which showed a similar accumulation of degree hours from January onwards (Figure 1). In line with the previous example, it suggests that the fluctuating vs. constant temperatures had no significant effect on the egg hatching of *L. dispar* or budburst timing of trees. Our results suggest that 6°C seems sufficient to accumulate chilling temperatures for temperate European trees, as it has recently been found experimentally (Baumgarten et al., 2021). In addition, the temperatures surrounding moth eggs depend on the females' choice of oviposition site, which can vary considerably: for example, under leaves, near the trunk section of trees or higher up the trunk (Kurenshchikov et al., 2020). Differences in height, shade, and albedo of the material where the eggs are deposited can greatly affect the microclimate (Ananko & Kolosov, 2021), making it difficult to predict egg hatching in future warmer conditions. In our study, microclimatic variation were avoided because the eggs were placed in plastic tubes protected from solar radiation, and the temperature was measured immediately adjacent to them. Our results therefore mimic the way in which increasing temperature could affect the phenology of this species in the absence of microclimatic effects.

Fourth, our study focused mainly on the temporal match between the first instar larvae of *L. dispar* and the emergence of leaves but not on the spatial match, which can be important because the larvae can disperse randomly by ballooning to other host trees, especially when they are starving (Foster et al., 2013), have poor‐quality food (Elkinton & Liebhold, 1990) or are on phenologically unsynchronized host trees (Erelli & Elkinton, 2000; Hunter & Elkinton, 2000). The ability of the *L. dispar* larvae to feed on suitable leaves may therefore depend on both temporal and spatial factors. However, modeling studies have shown that a lack of chilling during winter, resulting in a delay in budburst in temperate trees, can cause asynchrony between *L. dispar* egg hatching and leaf emergence over large spatial areas, such that dispersal by ballooning would not be sufficient to find suitable food (Foster et al., 2013). In addition, any dispersal attempt is associated with high mortality and is likely to be relevant only to the persistence of the species in a given area rather than to the success of an outbreak event.

Finally, natural enemies of *L. dispar* were not accounted for in this experimental study and may lead to complex interactions. For example, Hunter and Elkinton (2000) showed that the biomass gained by *L. dispar* decreases with leaf age, but survival may increase due to reduced predation pressure from natural enemies later in spring. The role of natural enemies for the fitness and performance of *L. dispar* on different tree species should be addressed in future studies.

## 5 CONCLUSION

Our experiment allowed us to simulate how the phenological synchronization between a generalist early‐spring feeding defoliating insect, the spongy moth *L. dispar*, and leaf emergence of common European tree species may change under warmer climates. The results demonstrated that some tree species (*A. pseudoplatanus*, *T. cordata*, and *F. sylvatica*) have limited ability to advance their phenology in response to winter and spring warming, likely due to lack of chilling, while other species (*Q. robur* and *C. betulus*) were able to substantially advance their phenology without affecting their bud developmental success. The hatching of *L. dispar* was particularly synchronized with the leaf emergence of *Q. robur*, irrespective of the climatic conditions. Although first instar larvae preferred and benefited most from leaves of *F. sylvatica*, the phenological sensitivity of *F. sylvatica* to chilling and photoperiod led to a clear phenological mismatch under warmer conditions. Our results suggest that increasing the proportion of oak in beech stands, as is currently being promoted in central Europe to increase forest resilience to climate change, may increase both the spatial and temporal window of host suitability for the spongy moth and lead to potential outbreaks.

## AUTHOR CONTRIBUTIONS

**Yann Vitasse:** Conceptualization (lead); formal analysis (lead); funding acquisition (lead); investigation (equal); methodology (lead); project administration (lead); supervision (lead); writing – original draft (lead); writing – review and editing (lead). **Nora Pohl:** Data curation (lead); investigation (lead); methodology (equal); writing – review and editing (supporting). **Manuel G. Walde:** Formal analysis (supporting); investigation (supporting); supervision (supporting); writing – review and editing (equal). **Hannah Nadel:** Methodology (supporting); resources (supporting); writing – review and editing (supporting). **Martin M. Gossner:** Conceptualization (supporting); methodology (supporting); writing – review and editing (equal). **Frederik Baumgarten:** Conceptualization (lead); investigation (equal); methodology (lead); supervision (lead); writing – original draft (supporting); writing – review and editing (equal).

## CONFLICT OF INTEREST STATEMENT

The authors declare no conflicts of interest.

## Supporting information


Data S1

## Data Availability

All data and R‐scripts used for the analyses are openly accessible via the digital repository platform EnviDat: https://doi.org/10.16904/envidat.419.
