# Supplementary material for: Feasting on the ordinary or starving for the exceptional in a warming climate: Phenological synchrony between spongy moth (Lymantria dispar) and budburst of six European tree species
Source: Ecol Evol. 2024 Feb 15;14(2):e10928. doi: 10.1002/ece3.10928 (PMC10869895; doi:10.1002/ece3.10928)
Supplement: Supplementary file 1 — Data S1 [file ECE3-14-e10928-s001.docx]

**Supplementary information**

Feasting on the ordinary or starving for the exceptional in a warming climate: phenological synchrony between spongy moth (*Lymantria dispar*) and budburst of six European tree species

**6 Tables and 9 Figures**

**Supplementary tables**

**Table S1.** Number of hatched larvae per treatment and egg mass identity

| Egg mass | Winter >10°C | Winter >6°C | OTC 3.5°C | OTC Ambient | Mean±SE | Sum |
| --- | --- | --- | --- | --- | --- | --- |
| 1 | 118 | 199 | 156 | 136 | 152.3±17.4 | 609 |
| 2 | 52 | 119 | 63 | 23 | 64.2±20.1 | 257 |
| 3 | 190 | 243 | 162 | 117 | 178±26.4 | 712 |
| 4 | 160 | 138 | 110 | 122 | 132.5±10.8 | 530 |
| 5 | 219 | 202 | 202 | 104 | 181.8±26.2 | 727 |
| TOTAL | 739 | 901 | 693 | 502 | 141.8±21.4 | 2835 |

**Table S2.** Growing degree hours (GDH) reached at the time of egg hatching for the US provenance used in experiment 1 and for the German provenance monitored in eight different sites in Switzerland by the Swiss rangers (see method) using either 0°C or 5°C as base temperature.

| Treatment / Site | Latitude  (DD) | Longitude  (DD) | Elevation (m) | GDD0  (°C hours) | GDD5  (°C hours) | Winter temperature (DJF, °C) | Provenance |
| --- | --- | --- | --- | --- | --- | --- | --- |
| OTC Ambient | 47.360 | 8.452 | 550 | 16’314 | 6’779 | 5.2 | US |
| OTC +3.5°C | 47.360 | 8.452 | 550 | 20’606 | 9’956 | 8.8 | US |
| Winter >6°C | 47.360 | 8.452 | 550 | 20’118 | 8’953 | 9.4 | US |
| Winter >10°C | 47.360 | 8.452 | 550 | 23’386 | 13’421 | 12.4 | US |
| Lombachalp | 46.750 | 7.903 | 1590 | 13’119 | 4’555 | -1.4 | Germany |
| Goldau | 47.049 | 8.553 | 514 | 14’387 | 5’370 | 2.2 | Germany |
| Pfaeffikersee | 47.353 | 8.769 | 570 | 13’864 | 4’947 | 2.3 | Germany |
| Selzach | 47.202 | 7.445 | 475 | 14’267 | 5’079 | 2.6 | Germany |
| Wildenstein | 47.434 | 7.749 | 565 | 13’789 | 3’271 | 2.8 | Germany |
| Reinach | 47.502 | 7.606 | 282 | 17’036 | 6’630 | 3.4 | Germany |
| Wiese | 47.576 | 7.623 | 266 | 17’705 | 6’961 | 3.5 | Germany |
| Lauterbrunnen | 46.717 | 7.929 | 580 | 15’702 | 5’344 | 4.5 | Germany |

**Table S3.** Summary of the analysis of variance performed on the hatching date depending on day of the year, temperature treatment, egg mass origin and the interaction between egg mass origin and temperature treatment, using a binomial family. Table shows chi square (χ2), degree of freedom (df) and *P*-value. Significant results (*P*<0.05) are indicated in bold.

|  | df | χ2 | *P*-value |
| --- | --- | --- | --- |
| DOY | 1 | 299.1 | **<0.001** |
| Temperature treatment | 3 | 118.1 | **<0.001** |
| Egg mass | 4 | 9.4 | 0.052 |
| Temperature treatment x Egg mass | 12 | 6.4 | 0.893 |

**Table S4.** Summary of the analysis of variance performed on the budburst date of tree species excluding *A. pseudoplatanus* (too low survival in two treatments) depending on temperature treatment, species and the interaction between temperature treatment and species using mother tree as random intercept factor. Table shows chi square (χ2), degree of freedom (df) and *P*-value. Significant results (*P*<0.05) are indicated in bold.

|  | df | χ2 | *P*-value |
| --- | --- | --- | --- |
| Temperature treatment | 3 | 45.8 | **<0.001** |
| Species | 4 | 159.4 | **<0.001** |
| Temperature x species | 12 | 41.7 | **<0.001** |

**Table S5.** Summary of the analysis of variance performed on the budburst date/hatching date depending on temperature treatment, species and the interaction between temperature treatment and species using mother tree/egg mass origin as random factor. Table shows chi square (χ2), degree of freedom (df) and *P*-value. Significant results (*P*<0.05) are indicated in bold.

|  | df | χ2 | *P*-value |
| --- | --- | --- | --- |
| Temperature treatment | 3 | 4096.1 | **<0.001** |
| Species | 5 | 176.6 | **<0.001** |
| Temperature x species | 15 | 67.6 | **<0.001** |

**Table S6.** Fresh biomass per larva (Mean±SE) and mortality rate (%+SE) after 1 week of feeding on young leaves of each study tree species

| Tree species leaves (1 week) | Fresh biomass per larva (mg) | Mortality rate (%) |
| --- | --- | --- |
| Control (no leaves 24h) | 0.48±0.03 | 0±0 |
| *Ulmus glabra* | 0.75±0.07 | 24±7.5 |
| *Acer pseudoplatanus* | 1.14±0.19 | 36±7.5 |
| *Tilia cordata* | 1.35±0.17 | 24±9.8 |
| *Carpinus betulus* | 3.48±0.70 | 20±6.32 |
| *Quercus robur* | 8.26±1.03 | 0±0 |
| *Fagus sylvatica* | 13.08±0.64 | 4±4.0 |

**Supplementary figures**


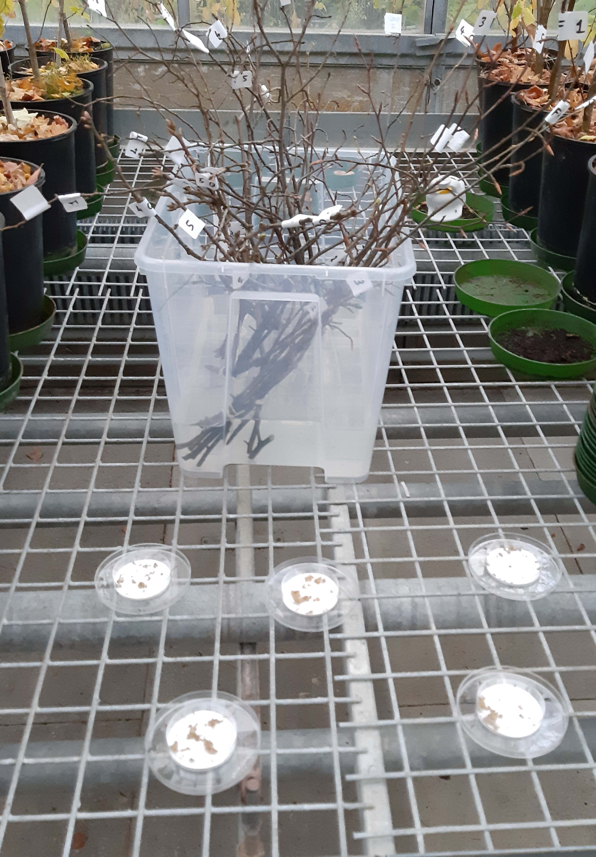

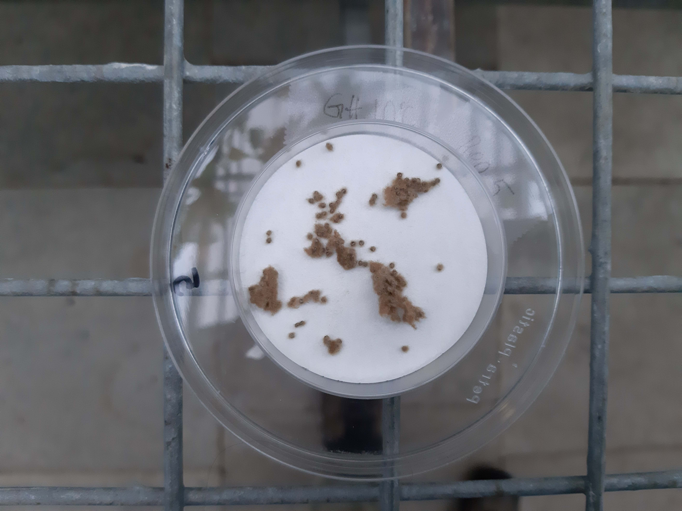


**Fig. S1.** Egg masses and twigs installed in the different climatic treatments before hatch/budburst. Twigs were pruned to a length of c. 50 cm and placed into plastic boxes with deionized water to minimize bacterial development. Eggs of *L. dispar* were placed in open petri-dishes that were positioned within a bigger one filled with a detergent to stop the larvae from escaping.

**Fig. S2.** Amount of chilling accumulated in the different treatments under different methods of calculation. a, Chilling portions accumulated according to the dynamic model (Luedeling and Gassner 2012); b, Chilling hours accumulated according to (Weinberger 1950); c, Chilling units accumulated according to the Utah model (Richardson 1974). Computation of the different chilling accumulation made by the ChillR package (Luedeling and Gassner 2012).


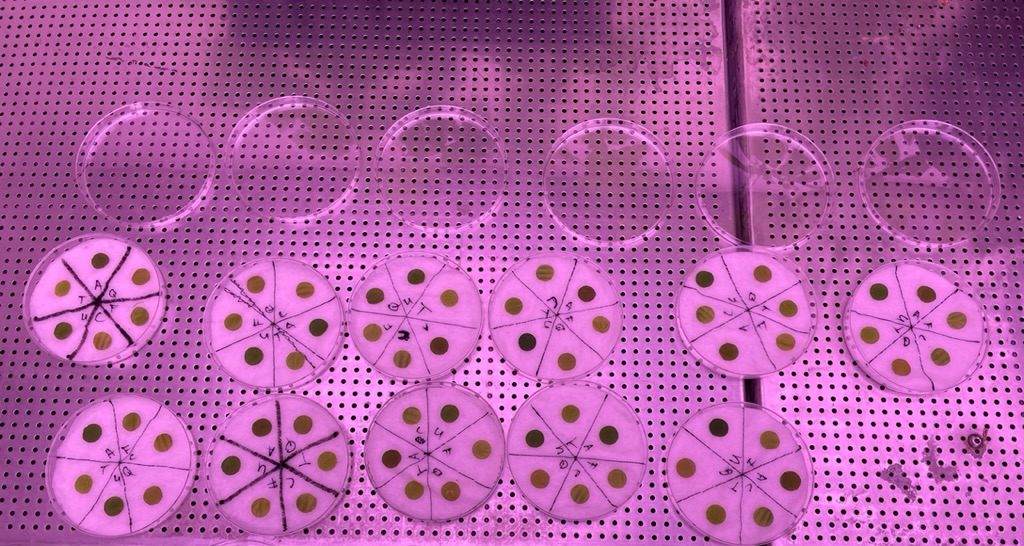


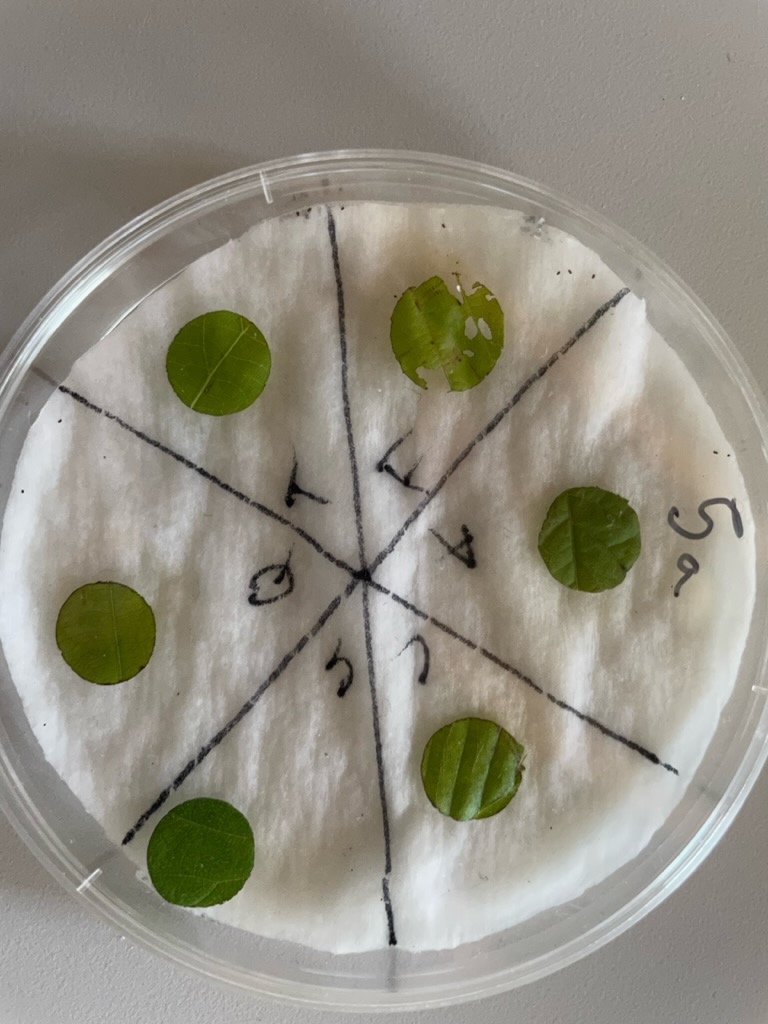

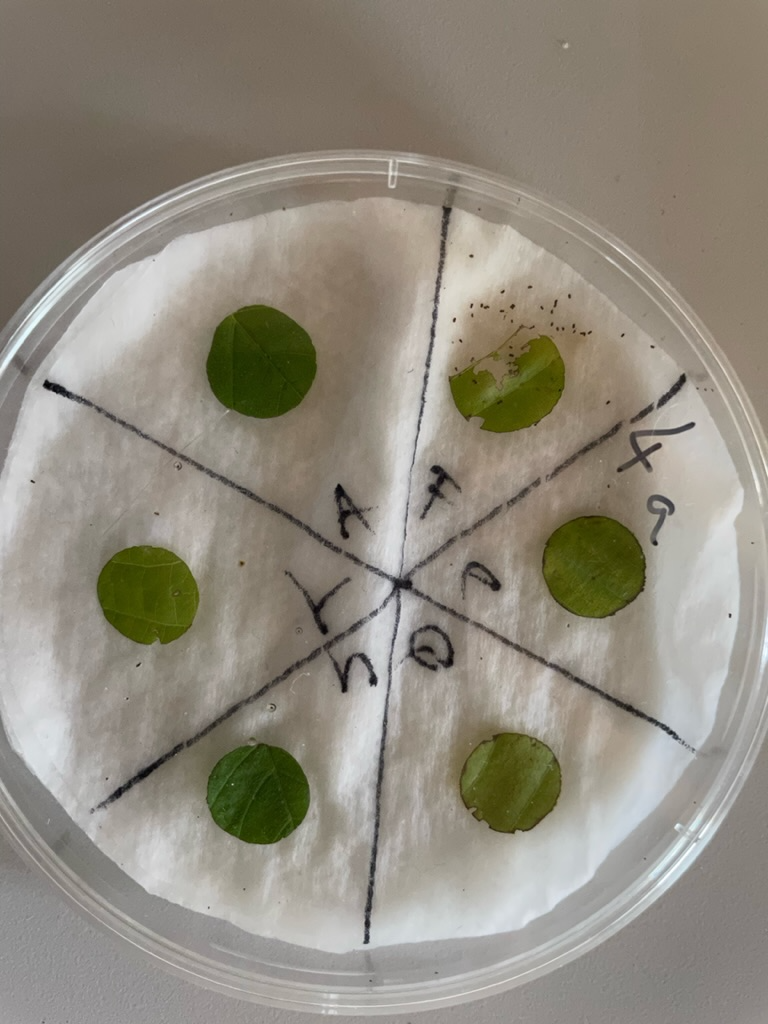

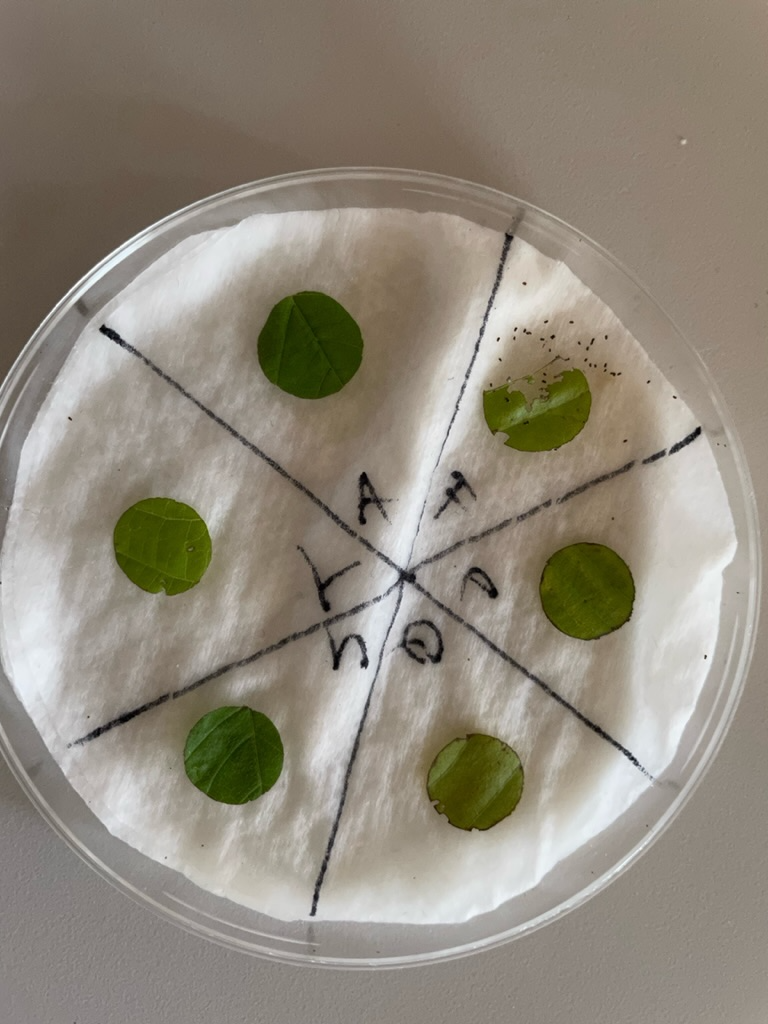

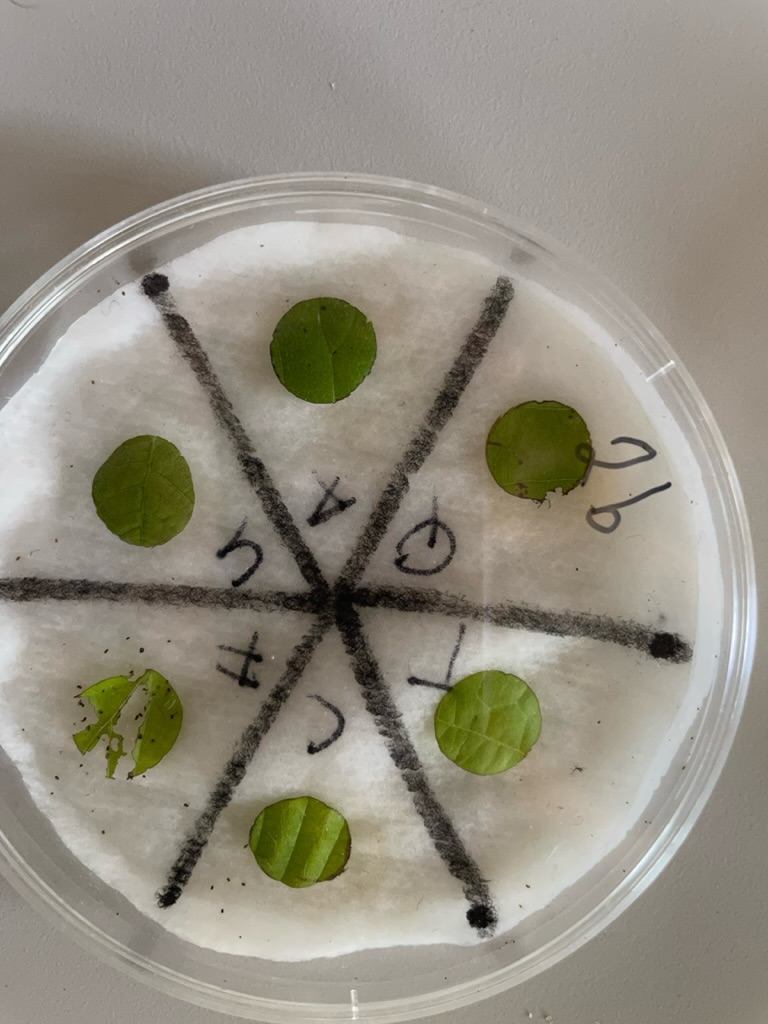

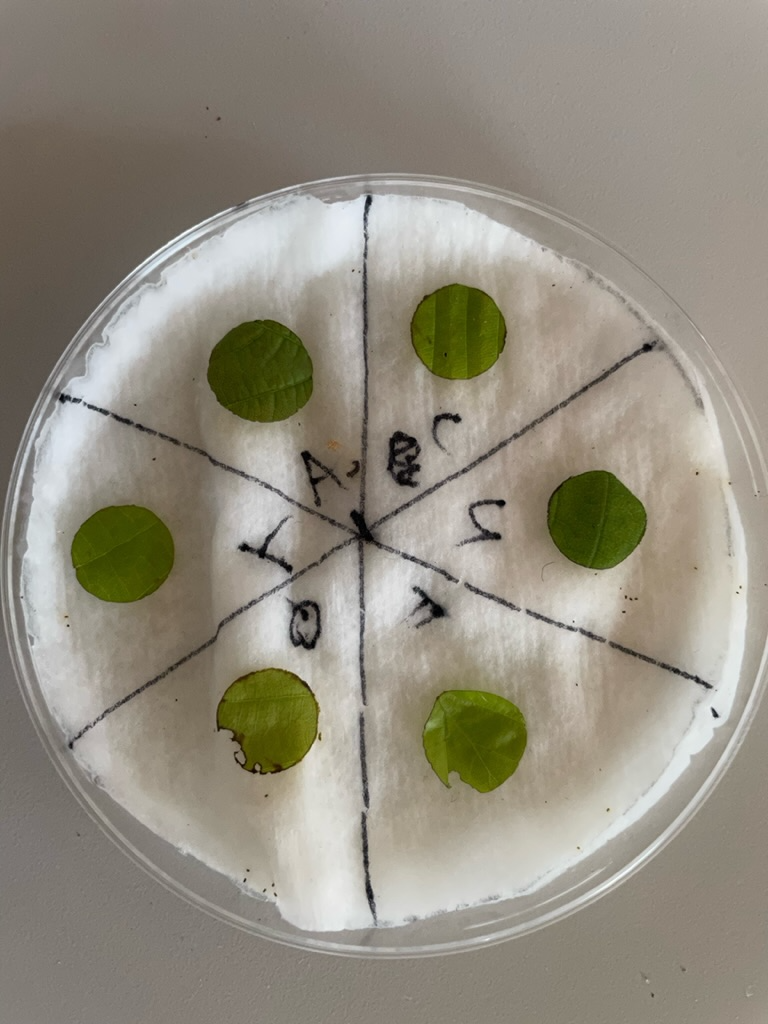

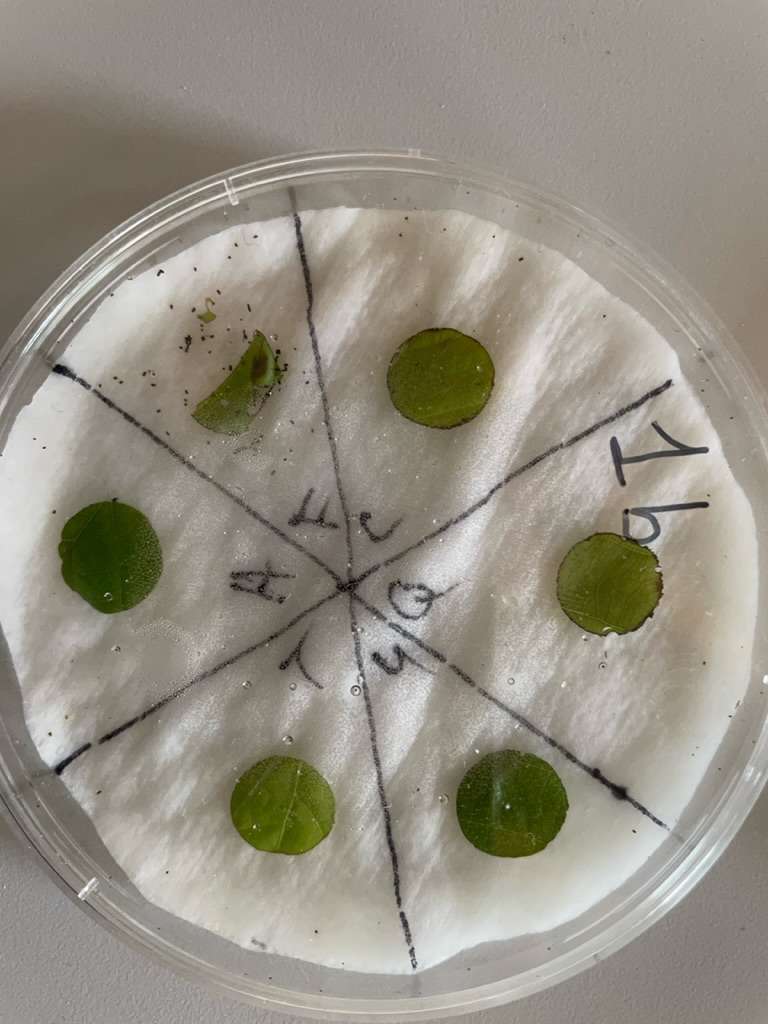


**Fig. S3.** Preference tests. Discs of 1.15 cm^2^ harvested from fresh young leaves of each study species were arranged in random order on wet absorbent paper in a petri dish with five first instar larvae placed in the middle of the petri dish for 24h at 20°C (upper panel). Lower panels show the discs after the experiment (A, *Acer pseudoplatanus*; T, *Tilia cordata*; F, *Fagus sylvatica*; U, *Ulmus glabra*; Q, *Quercus robur*; C, *Carpinus betulus*).


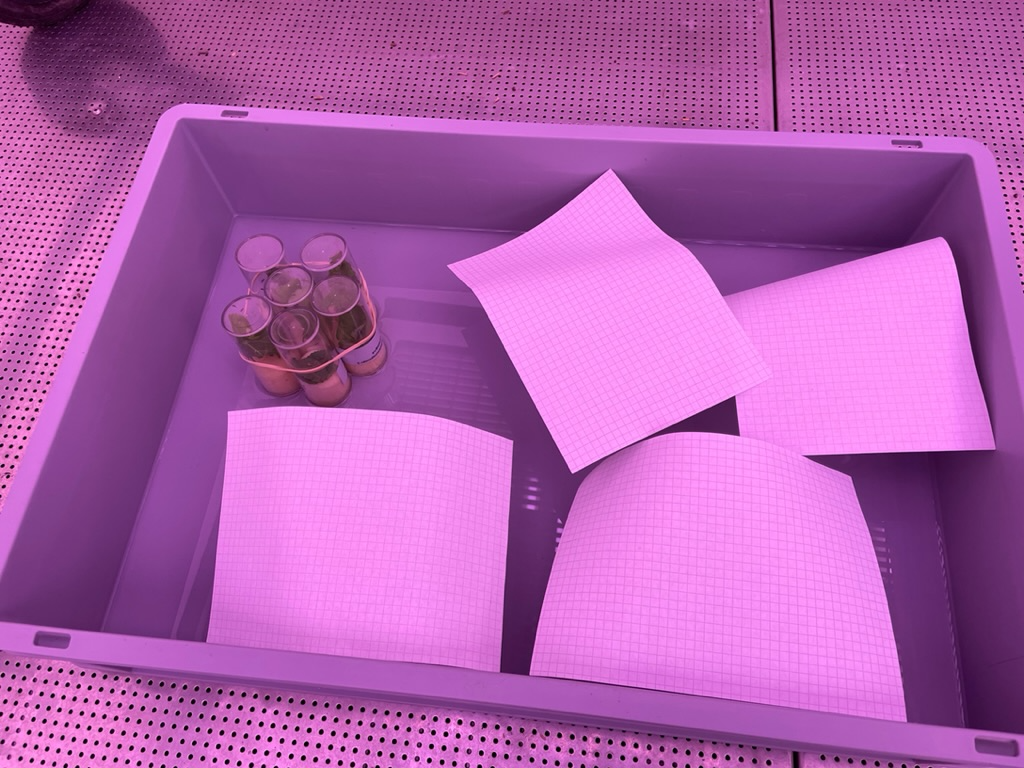

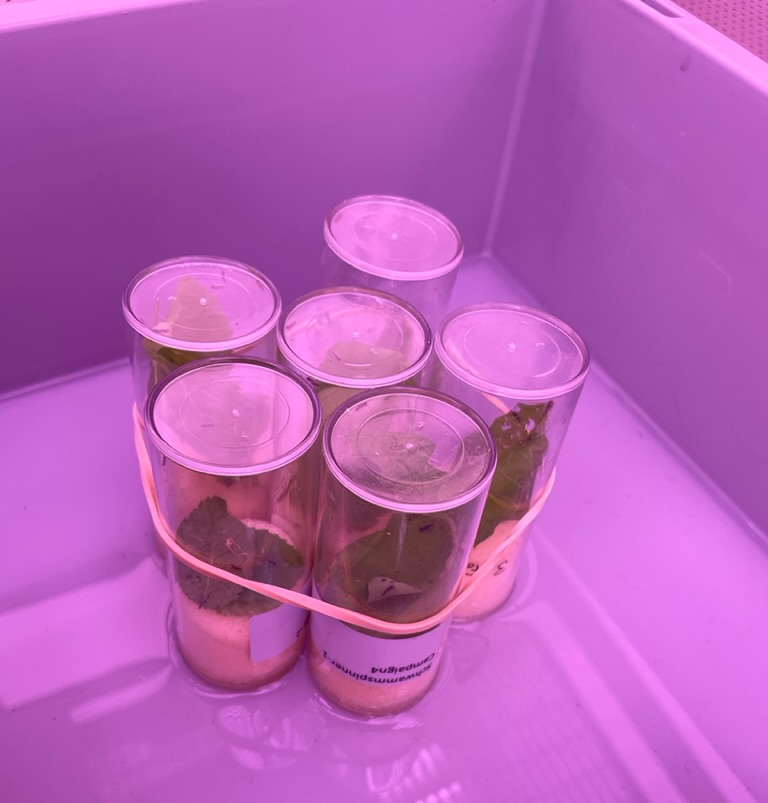


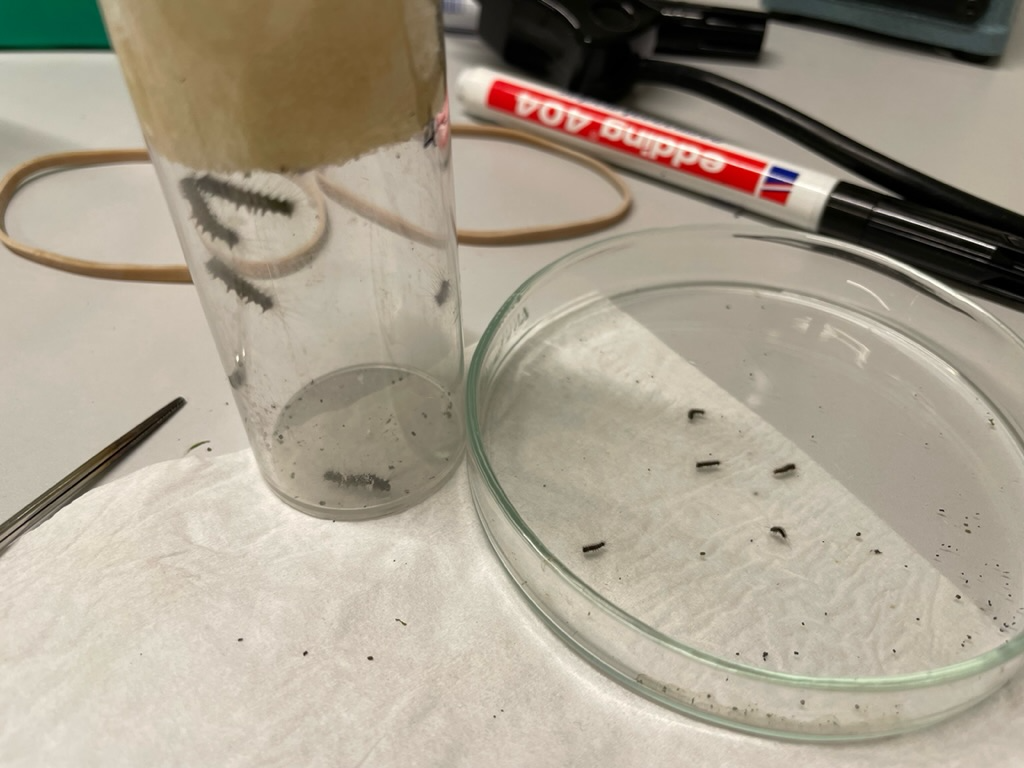


**Fig. S4.** Performance tests. Five first instar larvae (0–2 days after hatching without any food) from each egg mass source were kept in a plastic tube with a permeable foam cap filled with fresh young leaves of each tree species studied for one week at 20°C (upper panels). Tubes were put downside into a box filled with some water and a paper sheet covered the tubes to avoid excessive light intensity. Lower panel shows an example of one replicate containing five first instar larvae after one week fed with *F. sylvatica* leaves (left side of the picture, still in the tube) or *A. pseudoplatanus* leaves (right side, in the petri dish). Note the substantial difference in body size.

**Fig. S5.** Relationship between winter temperature and growing degree hours reached at the time of hatching for *L. dispar* eggs of the US provenance (orange) used in experiment 1 in the four different treatments and the German provenance (blue) used in experiment 2 overwintering in eight different forests in Switzerland (values are shown in Table S2).

**Fig. S6.** Hatch of *L. dispar* over time in the different treatments. Each line represents the number of new hatched larvae monitored twice a week per treatment.

**Fig. S7.** Progress of egg hatch over time in the different treatments for each egg mass.

Egg hatching lines were predicted using generalized linear mixed effect models of the binomial family. Each dot corresponds to the accumulated percentage of hatched eggs per egg mass and treatments (observations). Shaded areas surrounding the lines correspond to 95%CI of the predicted values.

**Fig. S8.** Bumping chart representing how the phenological ranking of tree budburst dates changed under the different temperature treatments.

**Fig. S9.** Degree hours required to budburst (twigs) or hatching (spongy moth, *Lymantria dispar*) in the different treatments using either 0°C (a) or 5°C (b) as base temperature.

Values correspond to marginal means predicted from the linear mixed effect model for the growing degree hours (GDH) at the time of budburst (stage 2) for twigs or 50% hatch for *L. dispar* ±95%CI using donor tree or egg mass as random effect. Within species, means followed by a common letter are not significantly different by the Tukey test at the 5% level of significance. Siday tests were used for *A. pseudoplatanus* because only two treatments were available for this species. Note that the marginal means of *A. pseudoplatanus* under Winter >10°C and Winter >6°C conditions are not displayed due to insufficient survival (<50%).
